# Development, implementation, and evaluation of a novel guideline engine for pediatric patients with severe traumatic brain injury: a study protocol

**DOI:** 10.1186/s43058-020-00012-w

**Published:** 2020-02-25

**Authors:** Meagan R. Pilar, Enola K. Proctor, Jose A. Pineda

**Affiliations:** 1 Washington University in St. Louis, Brown School, One Brookings Drive, Campus Box 1196, St. Louis, MO 63130 USA; 2 Children’s Hospital Los Angeles/University of Southern California, Keck School of Medicine, 4650 Sunset Blvd, Los Angeles, CA 90027 USA

**Keywords:** Head trauma, Traumatic brain injury, Children, Pediatrics, Implementation strategies

## Abstract

**Background:**

Severe traumatic brain injury (TBI) is a leading cause of death and disability for children. The Brain Trauma Foundation released evidence-based guidelines, a series of recommendations regarding care for pediatric patients with severe TBI. Clinical evidence suggests that adoption of guideline-based care improves outcomes in patients with severe TBI. However, guideline implementation has not been systematic or consistent in clinical practice. There is also a lack of information about implementation strategies that are effective given the nature of severe TBI care and the complex environment in the intensive care unit (ICU). Novel technology-based strategies may be uniquely suited to the fast-paced, transdisciplinary care delivered in the ICU, but such strategies must be carefully developed and evaluated to prevent unintended consequences within the system of care. This challenge presents a unique opportunity for intervention to more appropriately implement guideline-based care for pediatric patients with severe TBI.

**Methods:**

This mixed-method study will develop a novel technology-based bedside guideline engine (the implementation strategy) to facilitate uptake of evidence-based guidelines (the intervention) for management of severe TBI. Group model building and systems dynamics will inform the guideline engine design, and bedside functionality will be initially assessed through patient simulation. Using the Promoting Action on Research Implementation in Health Services (PARIHS) framework, we will determine the feasibility of incorporating the guideline engine in the ICU. Study participants will include pediatric patients with severe TBI and providers at three trauma centers. Quantitative data will include measures of guideline engine acceptance and organizational readiness for change. Qualitative data will include semi-structured interviews from clinicians. We will test the feasibility of incorporating the guideline engine in “real life practice” in preparation for a future clinical trial that will assess clinical and implementation outcomes, including feasibility, acceptability, and adoption of the guideline engine.

**Discussion:**

This study will lead to the development and feasibility testing of an adaptable strategy for implementing guideline-based care for severe TBI, a strategy that meets the needs of individual critical care environments and patients. A future study will test the adaptability and impact of the bedside guideline engine in a randomized clinical trial.

Contributions to the literature

- Research has shown that the adoption of guideline-based care improves outcomes for patients with severe TBI. However, these guidelines have not been applied systematically in the intensive care unit clinical setting.
- Technology-based implementation strategies may be uniquely suited to the fast-paced and transdisciplinary approaches of the intensive care unit. Technology-based strategies may facilitate the timely, consistent, and sustained application of evidence-based care in routine practice.
- This study combines implementation science frameworks and systems science to develop an innovative implementation strategy, the bedside guideline engine. Our goal is to reduce the gap between evidence-based knowledge and actionable patient data to ultimately impact patient outcomes.

## Background

### Prevalence and treatment of TBI in children

An estimated 1.7 million Americans sustain severe traumatic brain injury (sTBI) annually, resulting in more than 50,000 deaths [1]. The majority of sTBI survivors must contend with considerable disabilities and financial burden [2]. In 2013, more than 640,000 children ages 0–14 visited the emergency department due to TBI, resulting in nearly 18,000 hospitalizations [3]. Ultimately, more than 2100 children die annually because of sTBI [4]. Young children are particularly at-risk due to falls, as well as motor vehicle accidents and assault [4, 5]. Not surprisingly, sTBI is a leading cause of death and disability in children in the USA [6], making it a significant public health problem.

In 2019 the Brain Trauma Foundation (BTF) released a third edition of guidelines for the acute medical management of sTBI in infants, children, and adolescents [7]. These recommendations center around the avoidance of secondary insults to the injured brain. Secondary insults are abnormal physiological states (e.g., increased intracranial hypertension, low blood pressure, and fever) that aggravate the primary injury (i.e., injury the brain sustains at the time of trauma). Secondary insults have been consistently associated with poor outcomes after sTBI. Consequently, the BTF guidelines provide evidence-based recommendations and best practices regarding treatment interventions and patient monitoring [7]. Multiple reports provide evidence that the adoption of guideline-based care improves mortality and functional outcome in patients with sTBI [8–13]. The Centers for Disease Control and Prevention studied the impact of the BTF guidelines and found that the implementation and widespread use could decrease deaths by 50% and result in economic savings for both sTBI patients (estimated $288 million) and society as a whole (estimated $3.8 billion) [14].

### Challenges for promoting TBI guidelines in practice

Despite the availability of evidence-based approaches for treating TBI, the implementation of guideline-based recommendations is limited, particularly in pediatric patients [9–11, 13, 15–19]. The guidelines have not been systematically applied in clinical practice, and compromised fidelity to guideline-based care results in large variability in care and outcomes [20, 21]. A growing body of literature describes the difficulties associated with implementation [22, 23]. For example, individual-level characteristics (e.g., physicians’ knowledge of current recommendations), external conditions (e.g., insufficient time or resources to implement guideline), and patient preferences against a guideline can each pose barriers for care [24–29].

Additional challenges for widespread use of guideline-based care include a lack of strategies that account for the complex, multilevel nature of sTBI care and medical staff [30, 31]. Medical teams caring for children with sTBI are comprised of providers with different levels of expertise, diverse backgrounds and, in many instances, limited experience working together as a cohesive team. This revolving team dynamic creates fragmented communication and less than optimal working conditions for shared decision-making across disciplines [32].

The dissemination of sTBI-related materials also challenges guideline-based pediatric critical care practice. Research has highlighted the difficulty for physicians in remaining up-to-date with current recommended practices and guidelines [33]. Unfortunately, deployment methods for guidelines typically include disseminating paper or electronic materials, which are either distributed to individual team members or available through a shared document archive. These dissemination methods are problematic, particularly in the intensive care environment because the resources are often not readily available when team members are communicating or making time-sensitive decisions at the bedside. The lack of effective dissemination and implementation strategies to scale up and sustain guideline-based care for children with sTBI represents an opportunity to improve care and outcomes. These strategies need to be tailored to individual patients and the unique environment in any given intensive care setting.

### Technology-based strategies in clinical care

Novel technology-based strategies may be uniquely suited to the fast-paced and transdisciplinary approaches of a clinical care setting like the intensive care unit (ICU). A recent summary by the National Cancer Institute emphasized the efficacy of technology-based strategies to improve care [34]. However, such technology-based strategies must be carefully developed and evaluated to prevent unintended consequences within the system of care [10, 11, 22]. Given such complexities, effective and sustained application of evidence-based guidelines will require newly designed, breakthrough implementation strategies that are applicable to real world ICU practice [10, 33, 35]. This challenge presents a unique opportunity for intervention to more appropriately disseminate and implement guideline-based care for pediatric patients with sTBI.

### Innovative implementation strategy for sTBI patients

Building on existing knowledge of pediatric sTBI, the proposed study will harness implementation and system dynamics sciences to inform the development of a patient-centered strategy to accelerate the adoption and sustained delivery of care based on the best available evidence. This study will develop and test the feasibility of a novel clinical implementation strategy—hereafter called the bedside guideline engine (BGE)—to facilitate timely and consistent delivery of evidence-based sTBI care. Table 1 summarizes current challenges for analyzing patient data, communicating between care team members, and making bedside time-sensitive decisions, while highlighting the advantages of the BGE [36–38].

**Table 1:** Advantages of BGE over current approaches for delivery of guideline-based sTBI care

Issue	Current Approach	BGE
Inefficient access to guideline content delays care	Requires accessing paper or electronic depository documents (time-consuming, inconsistent, and difficult)	Content is always present and highly visible on dedicated bedside device for immediate review
Indirect or absent link between content and patient data interferes with fidelity to guidelines	Linking content to patient data requires active effort by providers through multi-step processes	Guideline content is automatically linked to the patient’s momentary condition
Patient’s condition triggers alerts, but alerts are not linked to recommended care	Static guidelines: providers must identify alerts and link them to recommended care	Alerts automatically trigger content-based recommendations, which are displayed at the bedside alongside patient data
Information overload	- Care team members individually merge data from bedside monitor, medical record, and guideline content. - Access to patient physiology trends is often difficult or not available.	- Data from relevant sources is merged and automatically linked to guideline-based content. - High-resolution trends of patient physiology are available to facilitate data review and decision-making
No efficient method for ongoing evaluation of timeliness of therapy and fidelity to guidelines	- Clinical care is recorded in the medical record, which requires retrospective data collection (manual or semi-automated). - Timeliness of therapy is just an estimate.	- Automated data extraction for efficient audits of care - Tracks time-stamped team interventions and feedback

This technology-based implementation strategy will bolster consistent clinical decision-making by providing efficient access to actionable patient information, as well as guideline-based therapeutic options. The BGE will consist of (1) the Component Neuromonitoring System (CNS Monitor, Moberg Research, Ambler, PA), an FDA approved bedside device that displays relevant patient information and records therapeutic interventions and (2) a computer-readable clinical pathway based on the pediatric BTF sTBI guidelines [19]. It will incorporate features of implementation strategies associated positive impact on both clinical practice and patient outcomes, including point of care decision support and real time automatic provision of recommendations—rather than just clinical assessments [36, 37, 39]. The BGE will also allow workflow tracking and systematic evaluations of team behavior to better understand the factors that facilitate or disable sustainable implementation of evidence-based care. Ultimately, the BGE will empower providers with consistent knowledge of evidence-based recommendations, which will facilitate more reliable and expedited care. Importantly, the BGE will allow for adaptation of evidence-based recommendations to individual patient needs and clinical scenarios.

### Conceptual framework

The proposed research activities are based on the Promoting Action on Research Implementation in Health Services (PARIHS) framework, which describes how three key interacting elements—evidence, context, and facilitation—influence the successful implementation of evidence-based practices [40, 41]. Table 2 outlines how the PARIHS framework will inform the design and implementation of the BGE in the ICU.

**Table 2:** Timeline for designing and evaluating the BGE while including core element of the PARIHS framework

Year 1			Years 1–2
PARIHS core elements	Evidence	Context	Facilitation
Approach	BTF guidelines for the acute medical management of severe TBI in infants, children, and adolescents	Assessment of the ICU environment and culture	1. Incorporation of the BTF guideline content into a computerized pathway 2. Linking the pathway to patient data (completes the BGE) 3. Evaluation of BGE initial acceptance, perceived benefit, challenges to use, and adoption potential (informs BGE design and adaptation) 4. Feasibility testing in simulation environment and real world practice (informs refinement of the BGE and design of future clinical trial)
Baseline bedside practice data collection, contextual evaluation			Evaluation of unadapted and adapted technology

## Methods

The focus of this project is to develop and test the feasibility of a novel technology-based BGE to facilitate timely adherence to evidence-based guidelines and bolster consistent clinical decision-making. The team will develop and evaluate a technology-driven implementation strategy (the BGE) that facilitates the uptake of evidence-based guidelines for management of sTBI at the bedside (aim 1). We will then determine the feasibility of incorporating the BGE in the ICU environment (aim 2). This will include evaluation of the extent of adaptation of the BGE that is required at each of three participating ICUs. Feasibility testing will yield real world practice identification of site-specific barriers and facilitators to implementation of the BGE. More information for each step in will be described in the following section.

### Aim 1: Develop and evaluate a technology-driven implementation strategy (the BGE) that facilitates the uptake of evidence-based guidelines for management of sTBI at the bedside

#### Study population, subjects, and recruitment

In aim 1, we will develop a novel technology-based bedside BGE that will make guideline-based care easier for providers to access and implement in real time. Development will take place at three regional pediatric trauma centers, which provide care for the majority of sTBI patients in the state of Missouri. The BGE will be developed using an interdisciplinary team of providers and researchers who have experience in implementation and system dynamics science, clinical decision support tools, critical care, neurosurgery, and sTBI.

### Procedures, instruments, and design

#### Capturing the ICU environment and culture

Staff observations and interviews

To inform the development of the BGE, we will first capture the ICU environment and culture from each clinical site. Using staff interviews and ethnographic observations [42], we will describe organizational patterns (leadership, team composition, workflow) in participating ICUs.

Participatory group modeling

We will then use group model building and systems dynamics to inform the design of the BGE. This process will shift information gathering to a structured approach instead of more traditional focus group approaches [43]. Participatory group model building methods are based on community-based system dynamics (CBSD), a participatory method for involving communities (in this case health care providers) in the process of understanding and changing systems from the endogenous or feedback perspective of system dynamics [44]. The method will provide a visual representation of relationships that impact the BGE’s flow with input from physician leaders, nursing leaders, and bedside care providers.

Members of the research team will travel to all clinical sites to build knowledge around hospital functioning in each ICU (leadership, team composition, workflow for decision-making). The research team will convene multidisciplinary care teams at each collaborating site to conduct group model building workshops. This form of system dynamics method incorporates stakeholder group participation in modeling the system to fully understand its complexities [45, 46]. The workshop outputs will include causal models that outline barriers and feedback loops, which will enable visual representation of relationships and mechanisms important to successful uptake of the BGE [47]. The insights from this work will inform the BGE’s workflow design by delineating each ICU’s initial inertia and efficiency—factors that impact how the BGE will benefit local workflow [48–50].

#### Designing the implementation strategy: BGE development

The BGE will serve as an implementation strategy to enhance the adoption and sustained delivery of evidence-based clinical care for sTBI patients. It will be developed using the InfoMap Markup Language (IML), a novel tool that allows development of a computer-readable clinical pathway from a generic guideline. To reduce information overload and optimize alignment with clinical practice, BTF guideline IML content will be organized via Learning Management Systems principles [51]. The IML pathway will then be deployed into a dedicated personal computer using CarePath, a recently developed software platform that reads IML content and generates a graphic flowchart. The personal computer will be mounted next to the bedside Component Neuromonitoring System (CNS) monitor (Moberg Research, Ambler, PA), an FDA-approved multimodality bedside monitor for patients with brain injury. Moberg Research developed this technology with funding from the National Institutes of Health (National Institute of Nursing Research) and the Department of Defense.

With technical support from Moberg Research, we will adapt the IML technology to fit aspects of sTBI pathophysiology and treatment that are unique to the pediatric population. The Moberg CNS monitor allows high-resolution (0.56 Hz) prospective digital archival of vital signs that measure exposure to secondary insults. This software will generate a graphic flowchart and connects the BGE to patient physiology, nursing input, and instructional content while recording time-stamped nursing and physician interventions.

All output will be displayed on the CNS monitor, which continuously collects and displays the patient’s vital sign trends, including input from neuromonitoring devices (i.e., intracranial pressure monitor). By directly downloading vital signs from the bedside CNS monitor, the BGE will provide high-resolution trends instead of low-resolution data from hourly documentation in the medical record. The display will give clinicians real-time bedside access to patient assessments, thus facilitating access to more accurate data regarding injury progression and response to therapy [30]. Additionally, the display will provide recommendations for clinicians with the next step suggested by the patient’s pathophysiology, nursing input, and clinical knowledge from the BTF guidelines.

During the development of the BGE, we will use an adaptation of the Stages of Implementation Completion tool (SIC) to monitor and evaluate the completion of implementation activities, the length of time taken, and the proportion of activities completed [52]. Stages to be tracked are (1) incorporation of critical elements of the ICU environment and culture into the BGE design, (2) incorporation of the BTF guidelines into the CarePath pathway, and (3) linking of patient data and CarePath using the CNS monitor [52].

**Fig. 1:**
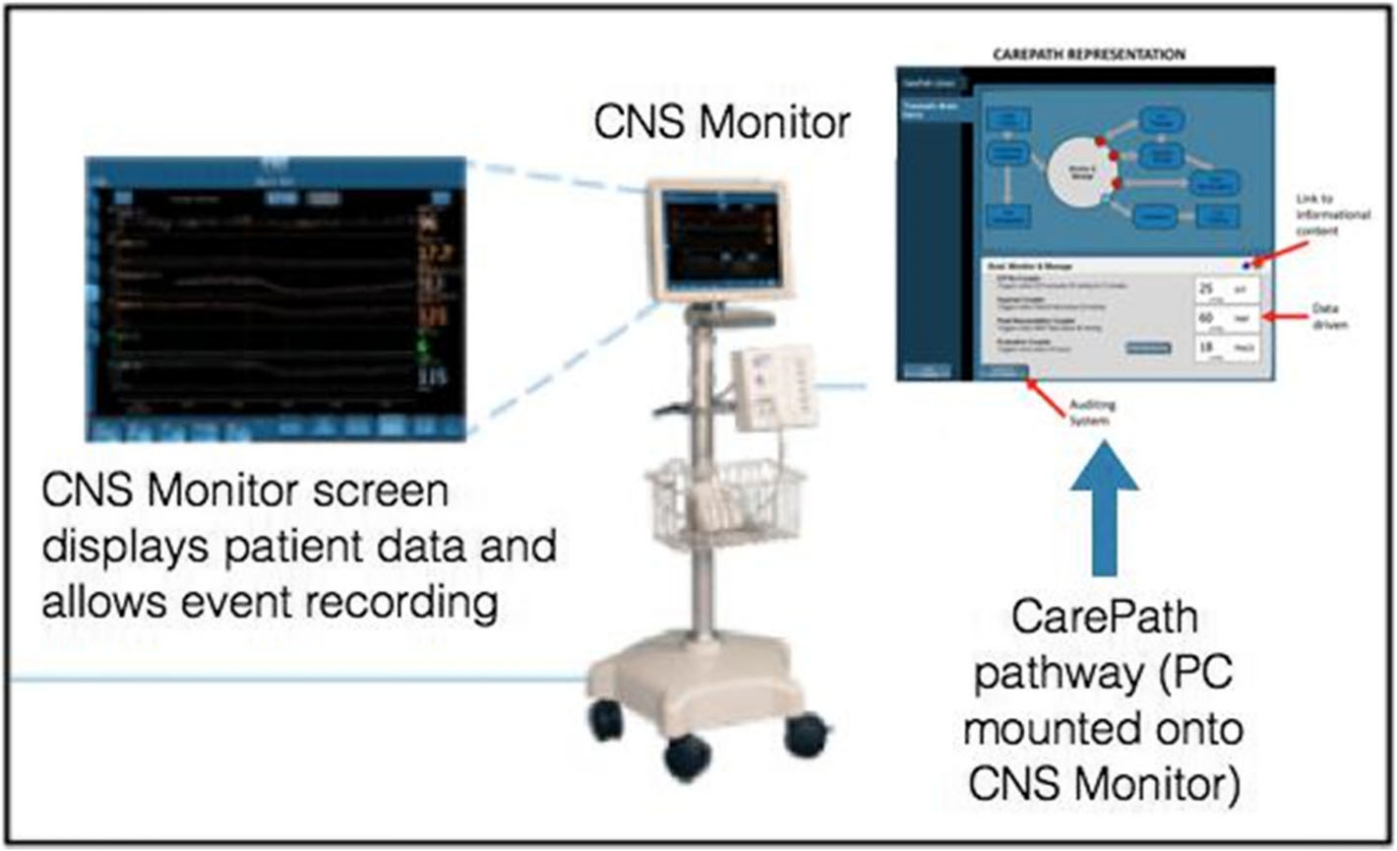
Example of CNS monitor display

It is important to note that the CNS monitor is not meant to substitute the clinician in the decision-making process. Instead, it is a tool for clinicians that is linked to patient pathophysiology and translates evidence-based guidelines into an electronic, interactive form available at the bedside. The BGE will track clinician-patient interactions and the patient’s response—or lack of response—to those interactions. Figure 1 provides an example of the BGE incorporated into the CNS monitor.

#### Evaluating the BGE’s initial acceptability

After the BGE has been designed, we will present it to ICU team members from all three sites and gauge initial BGE acceptance. Finally, we will use focus groups with ICU team members to assess acceptability and suggested improvements for the BGE [53]. These measures and processes will be further explained below.

#### Measures

Technology Acceptance Evaluation Model

We will evaluate acceptance of the technology-based BGE using a standardized technology acceptance questionnaire [54]. The questionnaire measures perceived ease of use, usefulness, and intention to use with seven-point Likert scales for all items.

Perceived Attributes of eHealth Innovations Questionnaire

We will evaluate the BGE’s acceptability using a 30-item questionnaire with demonstrated validity and reliability [55]. This measure will explore attributes of the BGE that may predict bedside adoption: relative advantage (better than existing solutions), compatibility (comports with adopters’ existing values and experiences), complexity/simplicity (relative difficulty or simplicity of use), trialability (can be used experimentally), and observability (results visible to outsiders). ICU team members using the BGE in their respective roles will complete a survey after training but before implementation in the patient simulation environment (*time point 1*).

#### Data collection and data analysis

##### Capturing the ICU environment and culture

Staff observations and interviews

Additionally, acceptance of the innovative BGE will be gauged by a collection of qualitative input from users [56, 57]. Data will be collected in separate sessions at each of the three clinical sites and will be analyzed using the grounded theory qualitative method [58].

##### Evaluating the BGE’s initial acceptability

Technology Acceptance Evaluation Model

All quantitative data will be managed and analyzed using the R statistical computation system [59]. As the questionnaire measures—perceived ease of use, efficacy, and intention to use/trust—have a skewed distribution, we specify an ordered logistic model [10]. The dataset also has a multiply-aggregated structure, as participants are nested within their group’s session, and sessions are nested within clinical sites. To address the violation of independent-observation assumption, we apply a three-level mixed model to the data analysis as follows:

$$ {\displaystyle \begin{array}{ccc}P& Q& R\\ {}{}^{ln}{\left({}^Y ijk\right)}^{=\gamma }{000}^{+}{\sum}^{\gamma }p00{\left({}^X\right)}_{pijk}& {}^{+}{\sum}^{\gamma }0q0{\left({}^S\right)}_{qjk}& {}^{+}{\sum}^{\gamma }00r\left({}^W\right){{{}_{rk}}^{+}}^u00{k}^{+r}0j{k}^{+e} ijk\\ {}p=1& q=1& r=1\end{array}} $$

where *ln(Y*_*ijk*_*)* is the outcome variable of interest for the *i*th participant working in the *j*th session from the *k*th site, *(X)*_*pijk*_ are *P* participant-level variables, *(S)*_*qjk*_ are *Q* session-level variables, *(W)*_*rk*_ are *R* site-level variables, *u*_*00k*_ is a random effect for the *k*^*th*^ site, *r*_*0jk*_ is a random effect for the *j*th session conducted in the *k*th site, and *e*_*ijk*_ is a residual term. Many individual-, session-, and site-level characteristics are coded as dichotomous variables. As such, the exponent of estimated coefficient exp (*γ*) for a dichotomous variable indicates percentage difference between groups on the level of technology acceptance. These effects, in conjunction with statistical significance testing, help discern barriers and facilitators of the implementation of BGE, which will be incorporated into the BGE’s design to optimize functionality and acceptance.

Perceived Attributes of eHealth Innovations Questionnaire

Responses will be aggregated to produce an overall assessment of the five criteria for design and adjustment purposes. Given the environment’s highly involved healthcare providers, we expect a high response rate and low missing data (e.g., refusals, “Do not Know” answers). Responses will be used to assess validity and reliability via confirmatory factor analysis on the five criteria, with exploratory factor analysis used to validate the contribution of individual questions. Table 4 in Atkinson 2007 [55] provides a general template for mapping specific questions to criteria. Cronbach’s alpha will test for internal consistency. The outcome will be environmental, ergonomic, and medical workflow information for design refinement.

Focus groups

Focus groups will include 5–8 team members of the same role (i.e., nurses, respiratory therapists). We will use principles of the Contextualized Technology Adaptation Process (CTAP) to guide the focus groups, as this framework considers multilevel influences on technology use and is specifically intended for technologies that are in the development or adaptation process [60]. In line with the CTAP, we will focus on the core domains of functionality (i.e., the range of operations provided by the technology) and presentation (i.e., the style of communicating information to the user), which provide a general structure for our sample stimulus questions.

These focus groups will allow users who have previously trialed the BGE to identify useful, irrelevant, and problematic features to inform refinement efforts [60]. Using a primarily deductive approach, we will conduct a directed content analysis by predetermining theoretically driven categories, coding themes based on those categories, and then forming new categories for responses to which the initial coding scheme could not be applied [61]. We will employ a QUAN → qual sequential triangulation, using qualitative feedback to explain and expand upon the quantitative findings derived from the questionnaire data captured from the standardized technology acceptance and perceived attributes of eHealth innovations questionnaires [54, 55]. The knowledge gained will inform adaptation of the BGE prior to evaluation in the patient simulation environment.

### Aim 2: Determine the feasibility of incorporating the BGE in the ICU environment

#### Study population, subjects, and recruitment

In aim 2, we will test the feasibility of incorporating the BGE into clinical practice. We will prospectively enroll 36 pediatric patients with sTBI in the ICU from the three proposed trauma centers (approximately 12 patients per clinical site) during the 24-month period. This sample size was selected based on feasibility and practical considerations, including the number of patients typically diagnosed with sTBI. Pediatric patients with sTBI (measured as a Glasgow Coma Scale score of < 8) admitted to the ICU at each clinical site will be screened for participation. Inclusion criteria for pediatric patients are as follows: (1) age 0–17 years, (2) accidental or non-accidental sTBI, and (3) placement of an intracranial monitoring device for clinical care. In the final stages of feasibility testing, written informed consent from the patient’s parents or legal guardian will be obtained by the research team before testing of the BGE, and safety measures will be in place to assure testing of the BGE does not compromise patient care. Given the low potential risk to human subjects and the challenges encountered when seeking written informed consent shortly after children are admitted to the ICU with sTBI, we will request the option of delayed written informed consent (up to 48 h) for cases when delaying consent is necessary (e.g., if parents are not reasonably available at the time of admission). For patients who choose not to participate in our feasibility testing, consent for prospective data collection from the medical record will be obtained. The data will be used for initial exploration of penetration of the BGE into clinical practice.

#### Procedures, instruments, and design

##### Feasibility testing: patient simulation

Once the BGE has been developed, we will introduce the BGE to a multidisciplinary group of ICU clinicians representing all clinical sites. We will use an adaptation of a previously described simulation module for trauma and critical care patients [62] to conduct the patient simulation using resources from the Sigh Pediatric Simulation Center at St. Louis Children’s Hospital, St. Louis, MO. We will then reapply the Perceived Attributes of eHealth Innovations questionnaire and the Technology Acceptance Evaluation Model (*time point 2*) and capture qualitative user input regarding the BGE in the simulation environment. This approach will inform evaluation and adjustment of the BGE and will assist with final configuration prior to bedside feasibility testing at the clinical sites.

Feasibility testing: clinical implementation

After being tested in a patient simulation environment and revised accordingly, we will conduct feasibility testing in our three clinical sites. Clinical implementation will be bolstered using an implementation facilitator (IF), as well as trainings for clinical staff.

Implementation facilitator

Feasibility testing at each clinical site will be conducted with assistance from a Pediatric Neurocritical Care Program implementation facilitator (IF). The IF will assure adequate dissemination of the knowledge needed for users to understand and use the tool. This will occur through the collaboration with local multidisciplinary stakeholders (i.e., each ICU’s physician and nursing leadership and bedside care providers) and tangible implementation goals with respect to uptake and fidelity. The goals include use of the BGE for bedside decision-making, including incorporation of evidence-based care recommendations. The IF will also lead the team as they set goals and work through the stages of the innovation decision process [63]. The incorporation of an IF has previously used when implementing new protocol and technologies [64]; however, it is still uncommon in acute care settings like the ICU. Providing expertise on implementation and dissemination will foster success and ease of adoption.

Training

Training will be provided to all users with initial emphasis on a subset of clinicians (charge nurses and ICU attendings), assuring a trained user will be available at all times to coach clinicians during the bedside feasibility testing. The IF, knowledgeable in the context and workflow of the participating ICUs, will work closely with the Washington University Dissemination and Implementation Research Core to apply a training program using a template developed by the State Implementation and Scaling-up of Evidence-based Practices (SISEP) Center at the University of North Carolina [65]. This approach emphasizes training and coaching on new skills that are necessary for effective use of innovations. Training will include videos developed in collaboration with Moberg ICU Solutions. The education process will continue until all providers are trained on BGE use, providing coaching, or advising and assisting through completion of the evaluation, to capture immediate feedback and increase fidelity [63, 64, 66, 67].

Clinician interviews

Finally, we will evaluate the BGE’s practicality (predicted cost, burden and benefit to ICU team), integration (extent of use and impact on other activities in the ICU), and expansion (perception of how expanded deployment would work) [68]. Information will be obtained through semi-structured interviews of clinicians (nurses, respiratory therapists, attending physicians and physicians in training) and hospital administrators [67, 69].

#### Data collection and data analysis

##### Feasibility testing: patient simulation

Perceived Attributes of eHealth Innovations Questionnaire

Data extracted will be analyzed using the same methodology as aim 1.

Cognitive interviewing

Use a think-aloud cognitive interviewing to obtain in vivo thoughts and reactions in the simulation environment [70].

##### Feasibility testing: clinical implementation

During the feasibility testing phase, the Technology Acceptance Evaluation Model and the Perceived Attributes of eHealth Innovations questionnaire will be distributed again [54, 55]. Similarly, cognitive interviewing will be used after implementation. Quantitative and qualitative data will be analyzed using the same methodology as previously described.

#### Trial status

Study procedures have been approved by the Institutional Review Board at Washington University in St. Louis and the University of Missouri, Kansas City. Data collection and BGE designing are ongoing at the time of submission of this manuscript (July 2019).

## Discussion

To improve clinical outcomes for children with sTBI, the best available evidence-based care must be more effectively tested and consistently applied in real-world practice. This study will develop and evaluate an innovative implementation strategy (a technology-based BGE) to facilitate timely delivery of evidence-based care of children with sTBI. The overall objective is to reduce fragmentation between evidence-based guideline information and actionable patient data, a barrier that typically stands in the way of fidelity to guidelines. If successful, this approach will also prove useful for implementation of guidelines for treatment of other complex and time sensitive conditions in the ICU environment.

Our innovative approach will incorporate state of the art technology and input from real world practice at three pediatric trauma centers. Our approach proposes significant change in the way care is delivered to children with sTBI by making evidence-based care easier for providers to apply in a timely fashion. The BGE will incorporate real-time feedback from the patient’s physiology through the CNS monitor while giving providers access to high resolution data trends; the BGE will then link these data to an evidence-based guideline with the goal of improving decision-making and timely delivery of therapy. The BGE will be built with capacity to efficiently incorporate new evidence as it becomes available. Ultimately, we anticipate this strategy will create a sustainable approach to efficient and consistent care for critically ill children with sTBI.

If successful, this approach may prove useful for the implementation of evidence-based guidelines in other settings where time sensitive, complex care is provided (e.g., other ICU conditions, the emergency department, or battlefield). Other ICU conditions that may benefit from this approach include management of nutrition, sedation/analgesia administration, treatment of life-threatening infections, and prevention of hospital acquired conditions. The innovation developed in this project may also contribute to the design of a future model of care that can adapt to different institutions and have a sustained impact on outcomes.

### Limitations

There are limitations to this project that should be noted. First, though previous and emerging evidence supports the effectiveness of guideline-based care in adults and children with sTBI, better understanding of the pathophysiology and effectiveness of specific interventions is needed. Additionally, in this 2-year project, it is possible that the number of patients in the feasibility testing phase will not be sufficient to adequately test the BGE. The participating adult trauma center ICU may have access to fewer pediatric patients, but since pediatric trauma patients can receive care at adult trauma centers, we believe it is important to gain knowledge from our feasibility testing in a non-pediatric trauma center/ICU environment. Given that we are using process-of-care outcomes as our metrics, we anticipate patient accrual will not be a significant limitation. Future studies are being planned to test the impact of the BGE on patient outcomes.

## Conclusions

Previous research has highlighted the need for an innovative implementation strategy to scale up and sustain guideline-based care. As such, we hope to use the BGE to improve outcomes in children with sTBI by facilitating timely, consistent, and sustained application of evidence-based care in routine practice. Our long-term goal is to develop and rigorously test an implementation strategy that fits the realities of clinical practice and individual patients and contributes to sustained implementation of guideline-based care for children with sTBI. This innovative proposal enables us to take the first step in this long-range implementation program.

Development of the BGE meets the broad need to decrease unnecessary variation in care and improve compliance with desired therapies, highlighting the relevance and generalizability of our approach to the wider ICU population [71]. We believe the proposed BGE will improve the application of adaptable guideline-based TBI care to meet the needs of individual ICU environments and patients. Additionally, this implementation strategy might guide the successful adaptation of our approach at other institutions. In a future comparative effectiveness clinical trial, we will propose scaled implementation of the BGE anticipating this approach will be superior to traditional care in translating knowledge into practice, positively impacting outcomes for children with sTBI.

## Data Availability

Data sharing is not applicable to this article as no datasets were generated or analyzed during the current study.

## Abbreviations

BGE: Bedside guideline engine
BTF: Brain Trauma Foundation
CBSD: Community-based system dynamics
CNS: Component Neuromonitoring System
CTAP: Contextualized Technology Adaptation Process
ICU: Intensive care unit
IF: Implementation facilitator
IML: InfoMap Markup Language
PARIHS: Promoting Action on Research Implementation in Health Services Framework
SIC: Stages of Implementation Completion
SISEP: State Implementation and Scaling-up of Evidence-based Practices
sTBI: Severe traumatic brain injury
TBI: Traumatic brain injury
